# Efficient propagation protocol and genetic similarity assessment of medicinal cannabis based on photoautotrophic micropropagation

**DOI:** 10.3389/fpls.2026.1846572

**Published:** 2026-06-10

**Authors:** Juwen Liang, Xiuye Wei, Jun Liu, Qing Zhou, Dongxian He

**Affiliations:** 1 College of Water Resources and Intelligence Engineering, China Agricultural University, Beijing, China; 2 Key Laboratory of Agricultural Engineering in Structure and Environment, Ministry of Agriculture and Rural Affairs, Beijing, China; 3 Institute of Chinese Materia Medica, China Academy of Chinese Medical Sciences, Beijing, China; 4 State Key Laboratory of Efficient Utilization of Agricultural Water Resources, China Agricultural University, Beijing, China

**Keywords:** *Cannabis sativa* L., ISSR markers, logistic growth model, plantlet, propagation coefficient

## Abstract

Photoautotrophic micropropagation (PAM), which employs sugar-free media and ventilated culture vessels to enhance plantlet photoautotrophic capacity and overall quality, has substantial potential to increase propagation efficiency when integrated with an appropriate propagation protocol. In this study, we established a PAM propagation protocol for medicinal cannabis (*Cannabis sativa* L.) using two genotypically different cultivars, ‘Charlotte’ and ‘Auto Charlotte’, and systematically evaluated the genetic similarity of plantlets across all successive batches via inter-simple sequence repeat (ISSR) markers as well as their performance after transplanting. Furthermore, a cultivar-specific logistic growth model was used to estimate annual yield and optimize the mother plant culture cycle for maximizing production. Results showed that under PAM conditions, mother plants sustained 11 batches of shoot tip harvest within a 98-d cycle. The propagation coefficients of ‘Charlotte’ and ‘Auto Charlotte’ reached 12.0 (95%CI: 11.0-13.0) and 11.3 (95%CI: 10.5-12.2), respectively. After 35 days of culture, plantlets from all batches exhibited uniform morphological and physiological traits, with rooting rates exceeding 70%. These plantlets could be directly transplanted without acclimatization, and minor growth differences between batches were eliminated by extending the culture cycle by one week. ISSR markers detected no obvious polymorphism between regenerated plantlets and their respective mother plants, with all similarity coefficients exceeding 0.90. Model simulations suggested that a 70-day culture cycle could maximize annual production, reaching a yield of 50,417–54,034 plants m^−2^. In conclusion, this study established an efficient PAM propagation protocol with high genetic similarity for the two tested medicinal cannabis cultivars, thereby providing technical support for cannabis plantlet production.

## 1 Introduction

Cannabis (*Cannabis sativa* L.) has emerged as an important economic crop due to its diverse applications in pharmaceuticals, nutraceuticals, and functional biomaterials. This species synthesizes more than 545 bioactive secondary metabolites, including at least 177 cannabinoids along with a wide variety of terpenes and flavonoids (Saragoca et al., 2025; Hanuš and Hod, 2020). Among these compounds, cannabidiol (CBD), cannabigerol (CBG), and various terpenoids have garnered considerable attention because of their pharmacological activities, including anti-inflammatory, analgesic, neuroprotective, and antiepileptic effects (Elsohly and Slade, 2005; Ben Amar, 2006; Nichols and Kaplan, 2020). Grand View Research predicts the global CBD market to reach USD 10.68 billion in 2025 and USD 22.05 billion by 2030, with a 15.8% compound annual growth rate (CAGR) (https://www.grandviewresearch.com/industry-analysis/cannabidiol-cbd-market, accessed on 11 November 2025). The rapid expansion of the medicinal cannabis market, particularly in the pharmaceutical sector, has imposed strict requirements on product quality, including batch-to-batch consistency and chemical uniformity. Accordingly, there is a growing demand for efficient and stable propagation protocols capable of producing genetically uniform, pathogen-free, and fully traceable female planting material.

In commercial production, cannabis propagation mainly relies on feminized seeds and cuttings, both of which present inherent limitations. As cannabis is dioecious with a highly heterozygous genome, seed propagation is prone to genetic segregation and chemotype instability, making it difficult to meet the stringent requirements for batch consistency and standardized cannabinoid content in pharmaceutical applications (O’Connell et al., 2017; Dreger et al., 2025). While cutting propagation preserves the maternal genotype, repeated harvesting facilitates pathogen accumulation in mother plants and increases susceptibility to pests and diseases. This ultimately leads to reduced vigor and inconsistent quality of planting materials (Hesami et al., 2023; Buirs and Punja, 2024). By contrast, photoautotrophic micropropagation (PAM) stands as a promising alternative to traditional propagation techniques for medicinal cannabis (Zarei et al., 2021; Shi et al., 2024). This technique employs sugar-free media within ventilated vessels to promote photosynthesis and transpiration, substantially increasing plantlet photoautotrophic capacity and overall growth performance. The elimination of exogenous carbohydrates substantially reduces bacterial and fungal contamination (Kozai and Xiao, 2008), while improved air exchange facilitates stomatal development and cuticle formation, enabling direct transplanting without acclimatization and achieving survival rates exceeding 90% (Kozai and Kubota, 2001; Park et al., 2011; Ashrafzadeh and Leung, 2021). Therefore, the application of PAM technology for the efficient propagation of medicinal cannabis has attracted growing interest in recent times.

Most existing studies on PAM of medicinal cannabis have focused on optimizing plantlet growth, rooting, and quality, encompassing aspects such as culture method exploration (Kodym and Leeb, 2019; Lubell-Brand et al., 2021), light environment optimization (Liang et al., 2025; Zarei et al., 2021), supporting materials selection (Ioannidis et al., 2022b), mineral element optimization (Zarei et al., 2023) and ventilation improvement (Murphy and Adelberg, 2021; Shi et al., 2024; Liang et al., 2026). In recent years, repeated shoot tip harvesting has been explored to enhance propagation efficiency for mass production of genetically homogeneous, chemically consistent, and pathogen-free planting material. For example, Lubell-Brand et al. (2021) achieved nearly 1, 800 shoot tips per square meter within 10 weeks, which was approximately nine times the yield of cutting propagation, although rooting success rates declined over time. Murphy and Adelberg (2021) reported multiple shoot tip harvests without subculturing, but agar collapse limited the process to four harvests. McKay et al. (2025a; 2025b) demonstrated that porous supporting materials supplemented with DKW liquid medium increase harvest frequency, and that supplemental far-red light enhanced shoot tip yield by 55%. Despite these advances, existing protocols remain constrained by high hormone dependence, agar collapse, and limited harvest batches, collectively leading to low propagation efficiency. Moreover, the controlled environment provides stable, ideal conditions for continuous propagation, yet mathematical models for estimating annual propagation yields are still lacking (Zheng et al., 2022).

However, long-term continuous subculture may induce somaclonal variation in cannabis, involving both genetic mutations and epigenetic modifications (Duta-Cornescu et al., 2023; Monthony et al., 2021). Factors such as explant origin, culture conditions, and the number of subculture cycles are reported to affect the incidence of such variation (Adhikary et al., 2021). Several studies employing inter-simple sequence repeat (ISSR) and other molecular markers have reported high genetic fidelity between plantlets and their mother plants (Dreger et al., 2025; Ioannidis et al., 2022a; Lata et al., 2010; Lata et al., 2016), which were consistent with findings that micropropagated plantlets show no differences in growth performance after transplanting and cannabinoid content compared to conventionally propagated plantlets (Kurtz et al., 2022). Nevertheless, some reports indicate that continuous subculture for 5–6 generations may alter the cannabis epigenome, potentially affecting gene expression and key agronomic traits (Adamek et al., 2023; Kastelec et al., 2025). Consequently, rigorous evaluation of plantlet genetic similarity and growth performance after transplanting is essential for establishing standardized annual propagation protocols.

This study aims to establish an annual propagation protocol for medicinal cannabis to improve plantlet propagation efficiency, providing technical support for production of all-female plantlets with high genetic similarity. Based on previously optimized lighting conditions, passive culture vessels, and CO_2_ enrichment strategies (Liang et al., 2025; Liang et al., 2026), we constructed a propagation system using porous supporting materials and nutrient replenishment to increase repeated harvest batches and shoot tip yield. We further assessed the genetic consistency between plantlets across different batches and evaluated their growth performance after transplanting. In addition, a yield estimation method based on the logistic growth model was established to optimize the mother plant culture cycle, thereby providing a quantitative basis for maximizing annual production and accurately formulating plantlet propagation schedules.

## 2 Materials and methods

### 2.1 Plant materials and culture conditions

#### 2.1.1 Source of donor plants

This study utilized two high-CBD medicinal cannabis cultivars sourced from Dutch Passion (Netherlands): ‘Charlotte’ (short-day) and ‘Auto Charlotte’ (day-neutral), both with THC < 0.3%, exhibiting CBD contents of approximately 15% and 10%-15%, respectively. Donor plants were cultivated following a previously established protocol (Liang et al., 2025) to ensure physiological consistency. The explants used in the experiment were shoot tips, excised from healthy, two-month-old female donor plants free of pests or diseases. Each explant, measuring 3.3 ± 0.3 cm in length and 1.9 ± 0.2 mm in stem diameter, possessed 1–2 fully expanded leaves, and had dry weights of 0.06 ± 0.02 g. Surface sterilization was performed by immersing the explants in 0.5% NaClO solution for 2 mins, followed by three rinses with sterile water. Subsequently, under aseptic conditions in a laminar flow hood, the basal end of each explant was trimmed at a 45° angle before inoculation.

#### 2.1.2 Culture vessels and supporting materials

Standard Magenta GA-7 vessels (dimensions: 75 × 75 × 100 mm, total volume 380 mL) were used. Each vessel was fitted with a lid perforated with two 8 mm-diameter circular holes, which were sealed with 18 mm hydrophobic fluoropore membranes (Milliseal, pore size 0.45 μm; Millipore, Japan) to enable air exchange. The air exchange rate of the vessels was quantified as 4.4 h^−1^ using CO_2_ as the tracer gas, with detailed methodological procedures described in our previous study (Liang et al., 2026). Each vessel was filled with approximately 9.0 g of sterile supporting material (peat: perlite: vermiculite = 1: 1: 1 v/v/v; bulk density: 0.15 g cm^−3^) and 45 mL half-strength MS medium containing 0.5 mg L^−1^ IBA. All prepared vessels were autoclaved at 121 °C for 20 minutes and then stored under aseptic conditions prior to inoculation.

#### 2.1.3 Culture conditions for mother plants and explants

Following inoculation, all vessels were placed on six-layer culture shelves (1200 × 600 × 1500 mm; 250 mm spacing between layers) with a density of 178 vessels m^−2^ in a controlled environment room. Consistent with our previous study (Liang et al., 2025), white LED lamps (W4000K-18W, Beijing Lighting Valley Technology Co., Ltd.) were installed 15 cm above the vessels as the sole light source (spectral distribution shown in **Supplementary Figure 1**). A photosynthetic photon flux density (PPFD) of 100-120 μmol m^−2^ s^−1^ was applied, with a photoperiod of 20 h d^−1^. Environmental parameters were controlled as: during light period, temperature, relative humidity (RH), and CO_2_ were maintained at 22 ± 1°C, 60% ± 5%, and 800 ± 50 μmol mol^−1^, respectively; during the dark period, these parameters were adjusted to 18 ± 1°C, and 70% ± 5%, while CO_2_ was not controlled.

#### 2.1.4 Growth conditions after transplanting

After 35 days of culture via PAM, uniform plantlets were transplanted into 6 L cultivation pots (top diameter 23 cm, height 21.5 cm, bottom diameter 18 cm). The cultivation substrate was consistent with PAM culture. Plantlets were irrigated daily with 100–250 mL of nutrient solution according to the plant size via a drip system. The solution exhibited an EC of 1.8 ± 0.2 mS cm^−1^ and a pH of 5.8 ± 0.2. It contained (in mg L^−1^): Ca(NO_3_)_2_·4H_2_O (865.7), KNO_3_ (316.4), NH_4_H_2_PO_4_ (111.3), (NH_4_)_2_SO_4_ (12.5), K_2_SO_4_ (118.1), MgSO_4_·7H_2_O (618.6), DTPA-Fe (40.0), Na_2_B_4_O_7_·10H_2_O (0.44), (NH_4_)_6_Mo_7_O_24_·4H_2_O (0.04), MnSO_4_·H_2_O (2.50), ZnSO_4_·7H_2_O (0.70), CuSO_4_·5H_2_O (0.16). The plantlets were cultivated under white LED lamps, with the canopy-level PPFD of 300-400 μmol m^−2^ s^−1^ and an 18 h d^−1^ photoperiod. Temperature and RH were controlled at 22 ± 1°C and 60% ± 5% during the light period, and adjusted to 18 ± 1°C and 70% ± 5% in the dark period; CO_2_ was not controlled during either period.

### 2.2 Experimental design

**Figure 1:**
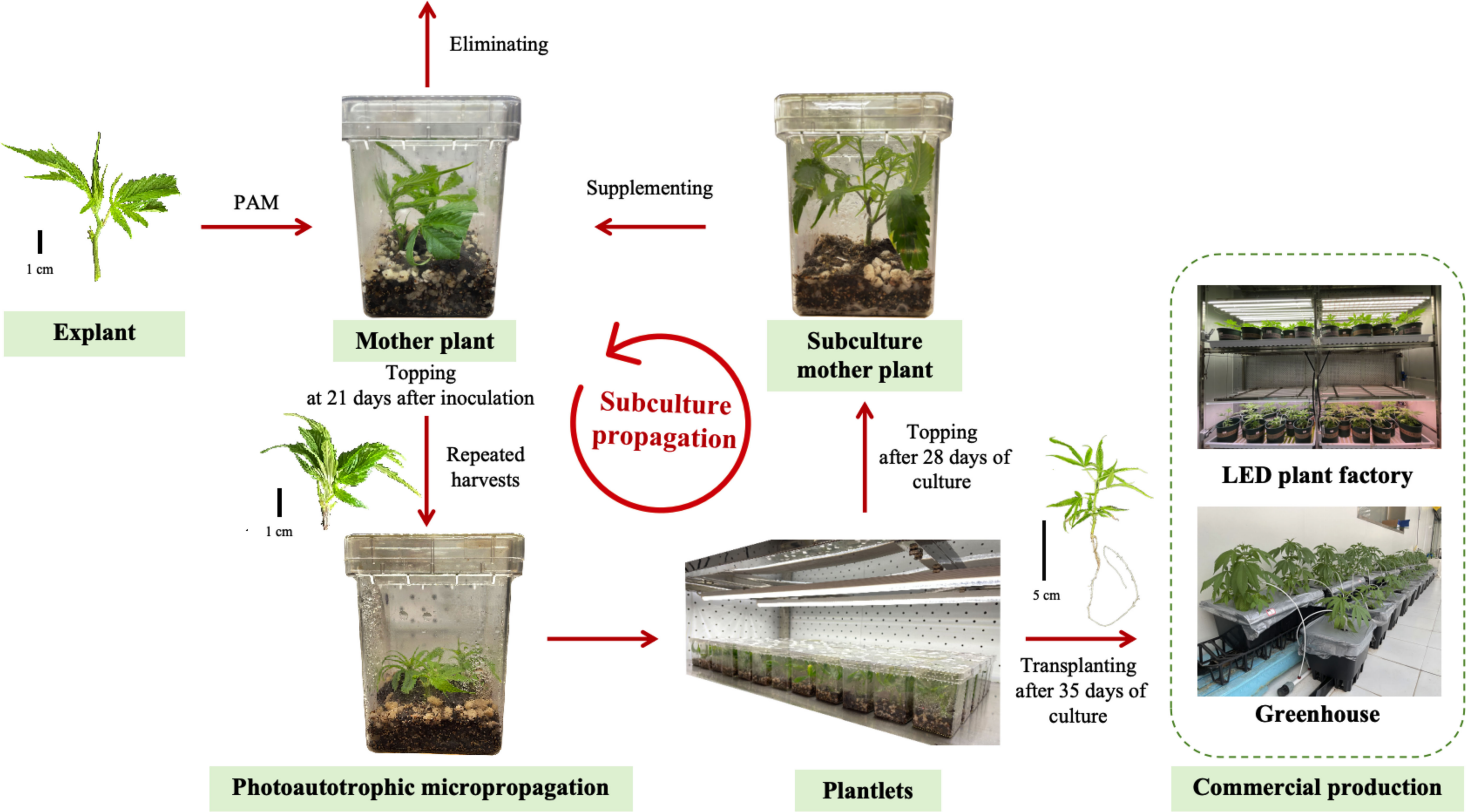
Propagation process for medicinal cannabis based on photoautotrophic micropropagation.

The PAM procedure for medicinal cannabis is illustrated in **Figure 1**. Female donor plants cultivated in an LED plant factory for more than two months were used as explant sources. Healthy, pest- and disease-free shoot tips were excised and trimmed to 2–3 cm to serve as explants for initial mother plant establishment. To optimize mother plant inoculation density, three density treatments were set: 1 (MP1), 2 (MP2), and 3 (MP3) plants per vessel, resulting in six treatment combinations (2 cultivars × 3 densities) with 12 vessels per treatment and two independent experimental repetitions. The second repetition was carried out under identical culture environments. After 21 days of culture via PAM, mother plants were topped to harvest the first batch of shoot tips, and 10 mL of hormone-free, half-strength MS liquid medium was added to promote axillary bud development. The second batch of shoot tips was harvested 14 days later, with another 10 mL of liquid medium added. Subsequent harvests were performed weekly, each supplemented with 5 mL of liquid medium. The number of shoot tip harvest batches (each batch corresponds to one round of shoot tip harvest) and shoot tip yield were recorded for each mother plant inoculation density treatment.

The harvested shoot tips were further cultured under PAM conditions. Specifically, these explants were grown for 28 days and then topped to serve as subculture mother plants for continuous shoot tip harvesting. Meanwhile, after 35 days of cultivation, plantlets derived from different harvesting batches were compared in terms of growth and morphological traits, as well as their genetic similarity to the original donor mother plants. The growth performance of plantlets from the 1st, 5th, and 11th batches was evaluated weekly within three weeks after transplanting, with four uniform plantlets selected per cultivar and batch for the assessment.

### 2.3 Measurements

#### 2.3.1 Logistic growth model fitting and annual propagation yield estimation

After each harvest, the cumulative number of shoot tips per vessel was calculated to quantify the propagation coefficient. This coefficient was defined as the total number of shoot tips harvested from a single mother plant over the entire culture cycle, with values determined by dividing the cumulative shoot tips yields by the initial number of inoculated shoot tips. The logistic growth model (Equation 1) fitted to the cumulative shoot tip production. To validate model reliability, parameters derived from the first trial were used to predict daily shoot tip cumulative number (*Y*) over a 98-day cultivation period, and predicted values were compared with measured values from the second trial. Model performance was assessed via the coefficient of determination (R^2^), root mean square error (RMSE), and mean absolute percentage error (MAPE).

(1) $$Y=\frac{Y_{M}\times Y_{0}}{\left((Y_{M}–Y_{0})\times \text{exp}(–\text{k}X)+Y_{0}\right)}$$

Where, *X* represents days after mother plant inoculation; *Y_0_*represents the number of shoot tips obtained from the first batch; *Y_M_* denotes the maximum shoot tip number per vessel within one culture cycle; k is the rate constant.

To simulate annual yield, the following parameters were assumed: the floor area of mother plant culture is 1 m^2^; all vessels were placed on a six-layer culture shelves, with a density of 178 vessels m^−2^, and two mother plants per vessel. The model was used to calculate the cumulative number of shoot tips harvested over 365 days, and the number of available commercial plantlets (assuming 80% rooting). Additionally, shoot tip harvest numbers and plantlet propagation numbers were calculated and compared across different mother plant culture cycles, including 63 days (6 harvest batches), 70 days (7 harvest batches), 77 days (8 harvest batches), 84 days (9 harvest batches), 91 days (10 harvest batches) and 98 days (11 harvest batches), to identify the optimal cultivation cycle for maximizing annual plantlet propagation yield.

#### 2.3.2 Measurement of plantlet growth

The harvested shoot tips were cultured via PAM. Three explants were inoculated per vessel, with a total of 12 vessels prepared for each batch of each cultivars. After 35 days of culture, four representative plantlets were randomly selected from each cultivar to assess their morphological indicators. Rooting rate was defined as the percentage of inoculated explants generating roots longer than 0.5 cm. Plantlet height and root length were determined with a ruler, and stem diameter using digital calipers. Internode and leaf numbers were counted manually. The SPAD value of the largest leaf was determined using a chlorophyll meter (SPAD-502, Konica Minolta, Tokyo, Japan), with five readings taken per plantlet and the mean value used for subsequent analysis. All leaves were detached from each plantlet, scanned using a flatbed scanner (LiDE400, Canon Inc., Tokyo, Japan), and their areas were quantified through image analysis with Adobe Photoshop CC 2019 (Adobe Systems Incorporated, San Jose, CA, USA).

#### 2.3.3 Growth indicator of plantlets after transplanting

After transplanting, the growth and morphological indicators of plantlets were measured weekly for three consecutive weeks. Plant height, canopy length (*L*), and canopy width (*W*) were obtained using a ruler, and the single plantlet canopy projected area (*A*) was calculated according to Equation 2. Stem diameter was determined using digital vernier calipers, and leaf number per plantlet was recorded manually. Four plantlets of each cultivar were selected for continuous tracking measurements.

(2) $$A=\frac{\pi }{4}\times L\times W$$

#### 2.3.4 Genetic similarity analysis using ISSR markers

Fresh leaves were collected from multiple individual plants of mother plants and 11 batches of plantlets of two cultivars. Each sample was ground and blended in liquid nitrogen, then stored at −80°C for subsequent experiments. Genomic DNA was extracted with a modified CTAB method (Xin, 2015). Six ISSR primer pairs reported to be suitable for cannabis by Xin (2015) and Adamek et al. (2023) were selected (**Supplementary Table 1**) and synthesized by Sangon Biotech (Shanghai, China). The PCR reactions were performed in a total volume of 25 μL, which comprised 12.5 μL of KOD One™ PCR Master Mix-Blue (Toyobo Biotechnology, Shanghai, China), 1 μL each of forward and reverse primers (10 μmol L^−1^), 1 μL of template DNA, and 9.5 μL of ddH_2_O. The PCR amplification procedure was set as follows: pre-denaturation at 98°C for 3 min, followed by 29 cycles of denaturation at 98°C for 10 s, annealing at 54°C for 5 s, and extension at 68°C for 3 s; final extension at 68°C for 10 min. Each sample was amplified in three independent PCR replicates. PCR products were resolved via 2% agarose gel electrophoresis at 180 V for 30–35 min, and visualized using a gel documentation system with DL2000 DNA Marker as reference. The presence and absence of amplified bands were recorded as binary data (1/0). Genetic diversity analysis was performed using POPGENE 1.32 software to evaluate genetic similarity.

### 2.4 Data analysis

Data were analyzed by one-way analysis of variance (ANOVA) with SPSS 26.0 software (IBM Corp., Armonk, NY, USA), and the significant differences among treatments were assessed via Duncan’s multiple range test at a significance level of *P* < 0.05. GraphPad Prism (v9.4, San Diego, CA, USA) was used for data visualization and curve-fitting. The logistic growth model was applied to fit the cumulative number of harvested shoot tips and the plantlet propagation number.

## 3 Results

### 3.1 Evaluation of medicinal cannabis propagated via the PAM protocol: yields and growth traits

**Figure 2:**
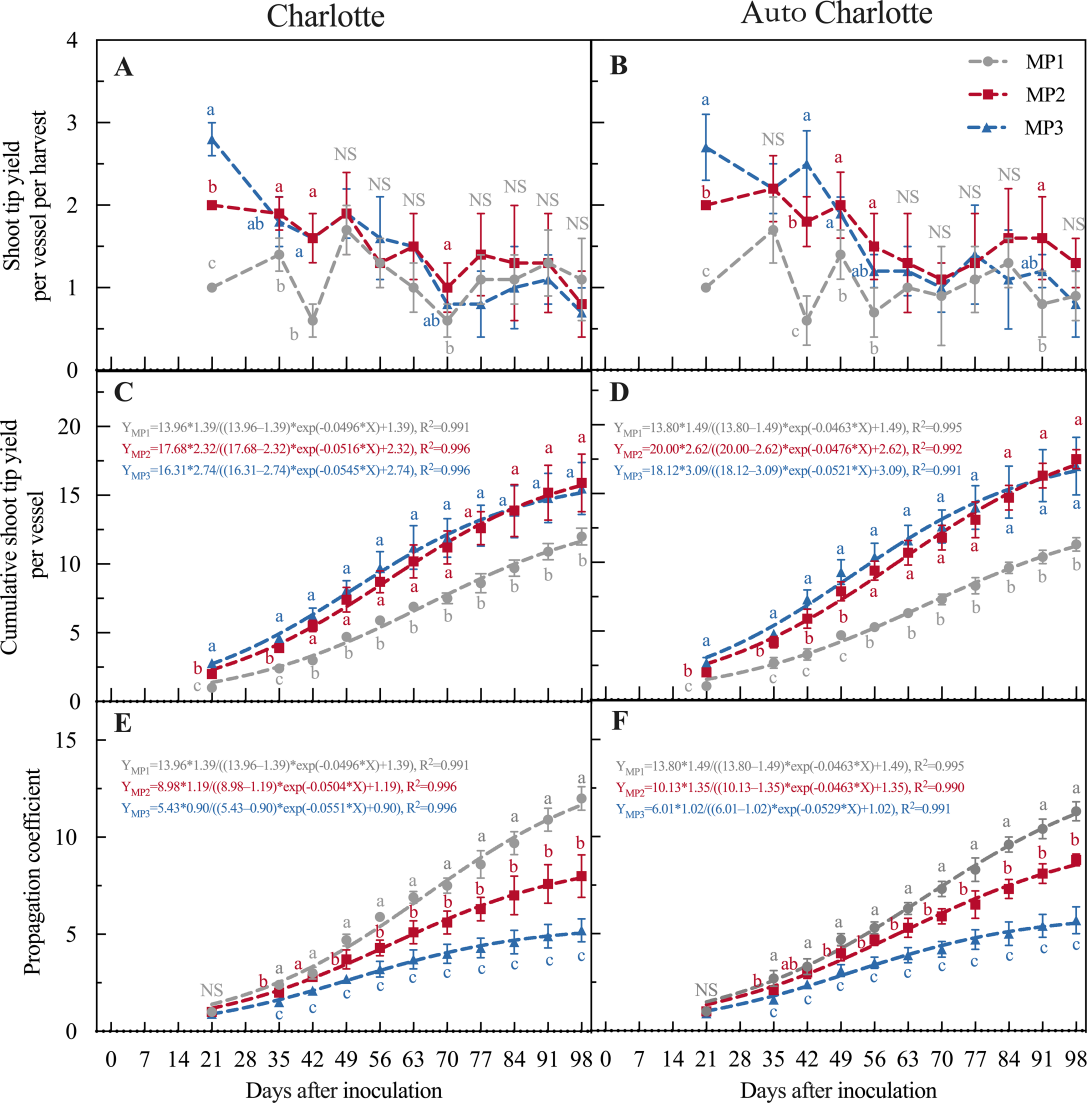
Effects of mother plant inoculation density on shoot tip yield. **(A, C, E)** Shoot tip yield per vessel per batch, cumulative shoot tip yield per vessel, and propagation coefficient of ‘Charlotte’; **(B, D, F)** The corresponding indicators of ‘Auto Charlotte’. MP1, MP2, and MP3 in the legend represent the number of mother plants inoculated per vessel. Both cumulative shoot tip yield per vessel and propagation coefficient were fitted using the logistic model. The propagation coefficient is defined as the cumulative number of shoot tips generated per mother plant. Different letters indicate significant differences (*P* < 0.05, n = 12). NS denotes no significant difference.

As the mother plant culture duration extended, the shoot tip yield per vessel per batch generally decreased for both cultivars (**Figures 2A, B**). During the early harvest stages, treatments MP2 and MP3 yield significantly more shoot tips per batch than MP1. In the later stages, while MP2 resulted in slightly higher yield, no significant differences were detected among the different treatments. ANOVA results confirmed that inoculation density had significant effects on shoot tip yield (**Supplementary Table 2**). For both cultivars, the cumulative shoot tips yield per vessel exhibited a typical sigmoidal growth pattern under all inoculation densities, and logistic models provided excellent fits (*R^2^* ≥ 0.99) (**Figures 2C, D**). During 1–4 weeks after the first batch, MP3 showed the highest cumulative shoot tip yield, significantly exceeding that of MP1. However, from weeks 9-11, cumulative yield in MP2 surpassed that in MP3, although the difference was not significant. By week 11, cumulative shoot tip yield per vessel in MP2 reached 15.9 for ‘Charlotte’ and 17.5 for ‘Auto Charlotte’. These values were 32.6% and 54.4% higher than MP1, respectively. Furthermore, propagation coefficient was negatively correlated with increasing inoculation density (**Figures 2E, F**), and as shown in **Supplementary Table 2**, inoculation density significantly affected this parameter throughout the entire culture period. Both ‘Charlotte’ and ‘Auto Charlotte’ achieved their highest propagation coefficients under MP1, reaching 12.0 (95%CI: 11.01-12.99) and 11.3 (95%CI: 10.48-12.17) respectively, whereas higher densities resulted in significantly reduction in this parameter.

**Figure 3:**
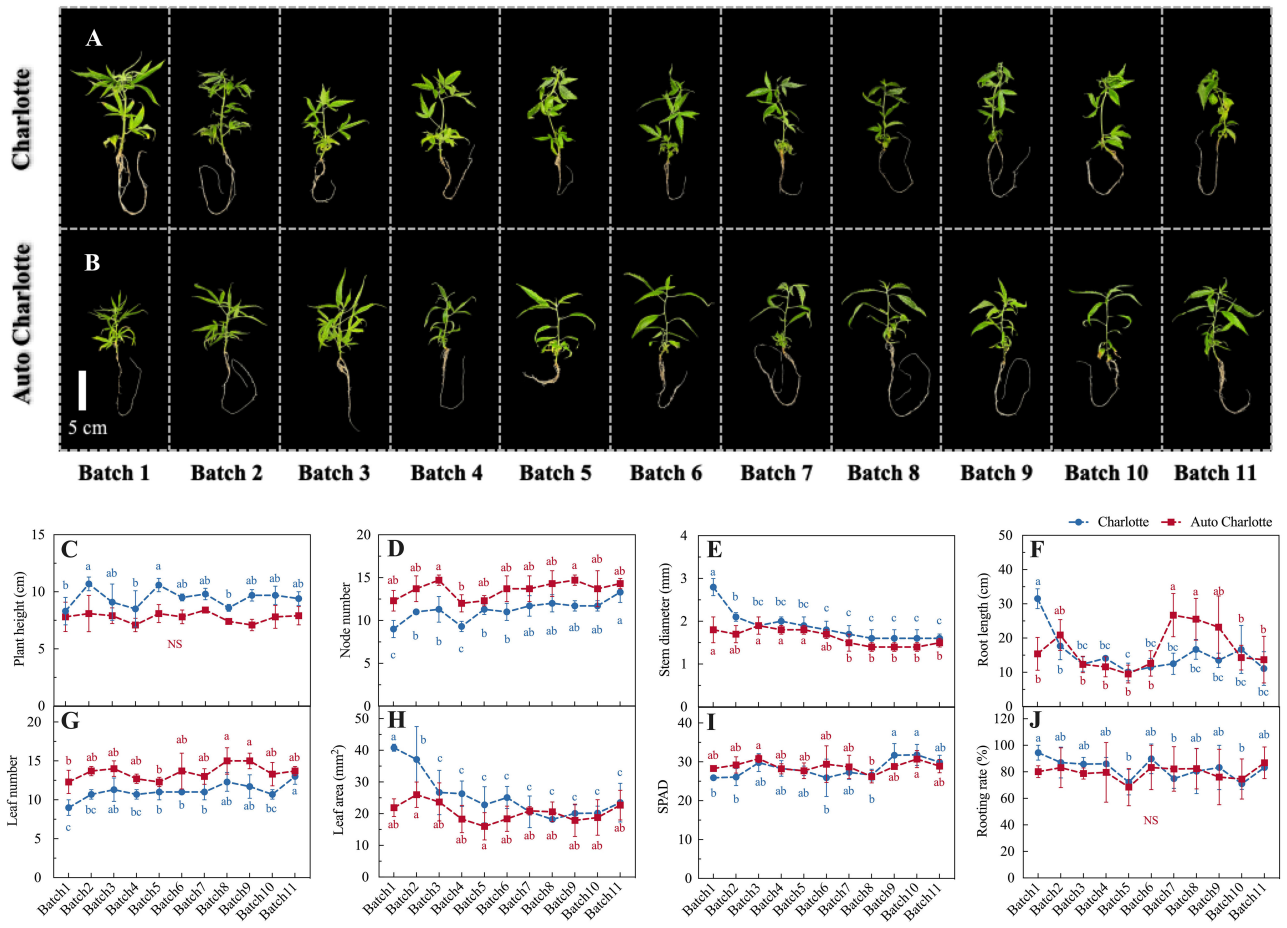
Representative photographs and morphological indicators of plantlets from 11 batches after 35 days of culture via PAM. **(A, B)** Overall growth performance of ‘Charlotte’ and ‘Auto Charlotte’ plantlets across the 11 batches, where numbers 1–11 represent the sequence of harvest batches. **(C)** Plant height, **(D)** node number, **(E)** stem diameter, **(F)** root length, **(G)** leaf number, **(H)** leaf area, **(I)** SPAD value, and **(J)** rooting rate of plantlets. Different letters indicate significant differences (*P* < 0.05, n = 4). NS denotes no significant difference.

The 11 batches of shoot tips harvested from both cultivars were cultured via PAM for 35 days, and their growth performance is summarized in **Figure 3**. For ‘Charlotte’, plantlets from the first batch had greater stem diameter, root length, and leaf area, with values of 2.8 mm, 31.5 cm, and 40.8 cm², respectively. These indexes were significantly higher than those of plantlets from batches 2-11 (**Figures 3E, F, H**). However, batches 2–11 showed no obvious increasing or decreasing trends in plant height, number of nodes, root length, number of leaves, leaf area, and SPAD value. This indicates stable growth performance across successive batches (**Figures 3C, D, F–I**). For ‘Auto Charlotte’, stem diameter gradually decreased across batches, from 1.8 mm in the first batch to 1.4 mm in batches 8 and 10, representing a reduction of 22.2% (**Figure 3E**). By contrast, the number of internodes and leaves gradually increased. The values rose from 12.3 internodes and 12.3 leaves in the first batch to 14.3 internodes and 13.7 leaves in the 11th batch. The corresponding increases rates were16.3% and 11.4%, respectively. Nevertheless, plant height, root length, leaf area, and SPAD values were relatively stable across all batches. In addition, the rooting rates of plantlets from all batches of both cultivars consistently exceeded 70% and showed no declining trend (**Figure 3J**).

**Figure 4:**
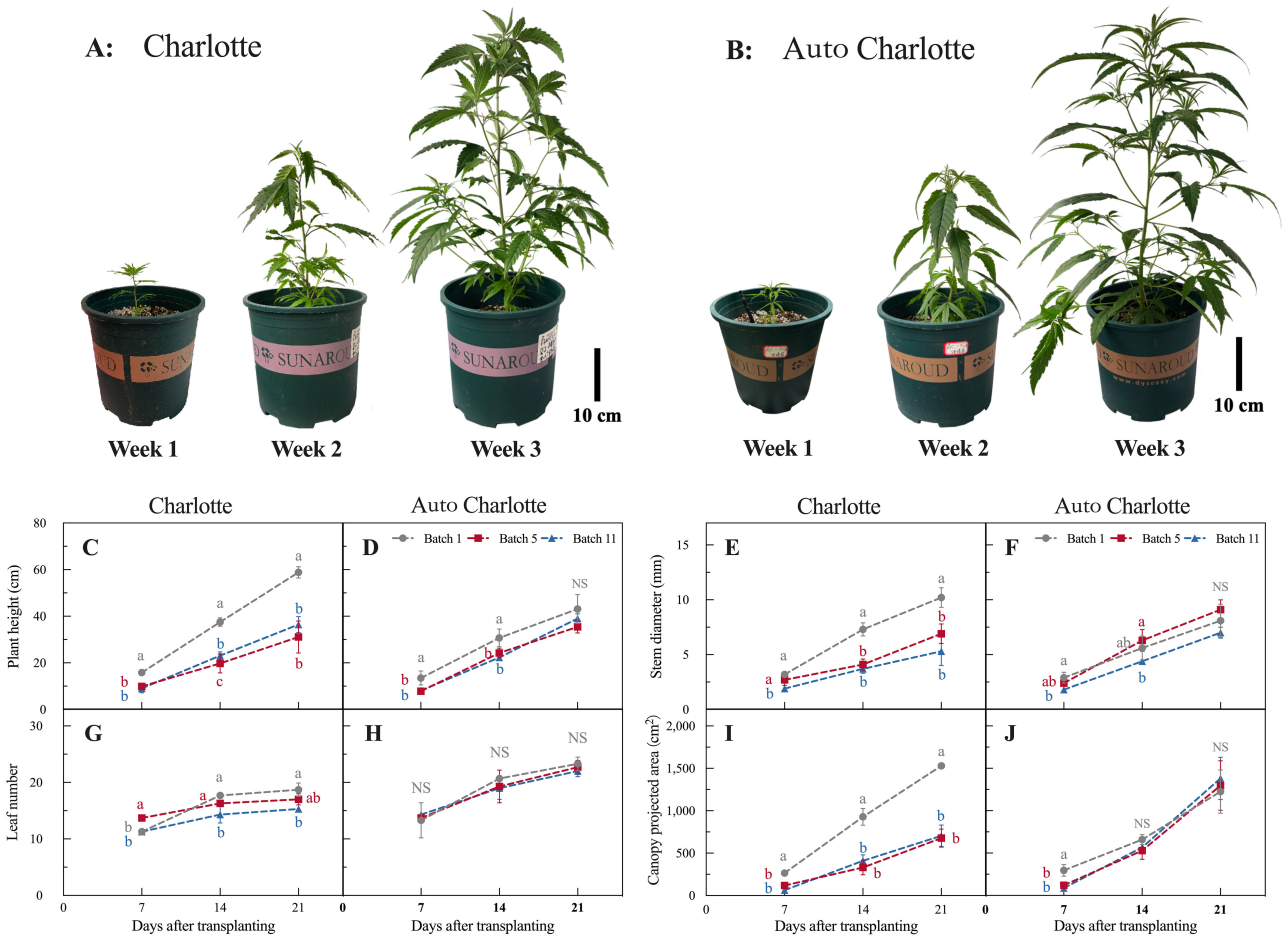
Growth performance of plantlets after transplanting. **(A, B)** The overall growth performance of plantlets from the first batch of ‘Charlotte’ and ‘Auto Charlotte’ at 1, 2, and 3 weeks after transplanting. **(C)** Plant height, **(E)** stem diameter, **(G)** leaf number, and **(I)** canopy projected area of ‘Charlotte’ plantlets from the 1st, 5th, and 11th batches; **(D, F, H, J)** the corresponding parameters for ‘Auto Charlotte’. Canopy projected area was calculated based on canopy length and width. Different letters indicate significant differences (*P* < 0.05, n = 4). NS denotes no significant difference.

After direct transplanting without acclimatization, plantlets of both cultivars exhibited vigorous growth and healthy foliage (**Figures 4A, B**). For ‘Charlotte’, the plant height, stem diameter, number of leaves, and canopy projection area of plantlets from the 5th and 11th batches were consistently lower than those of the 1st batch (**Figures 4C, E, G, I**). By the third week, the 1st batch reached 58.8 cm in height, 10.2 mm in stem diameter, 18.7 leaves, and a canopy area of 1530.2 cm^2^. The 11th batch only reached 36.4 cm, 5.3 mm, 15.3 leaves, and 704.4 cm^2^, with respective reductions of 38.1%, 48.0%, 18.2%, and 54.0%. These differences were probably attributed to the larger initial stem diameter, root length, and leaf area of plantlets in the first batch. Such disparities could be eliminated by extending the cultivation time of subsequent batches for one extra week. For ‘Auto Charlotte’, no significant differences remained among the 1st, 5th, and 11th batches by the 3rd week (**Figures 4D, F, H, J**).

### 3.2 Genetic similarity of plantlets across different batches

**Figure 5:**
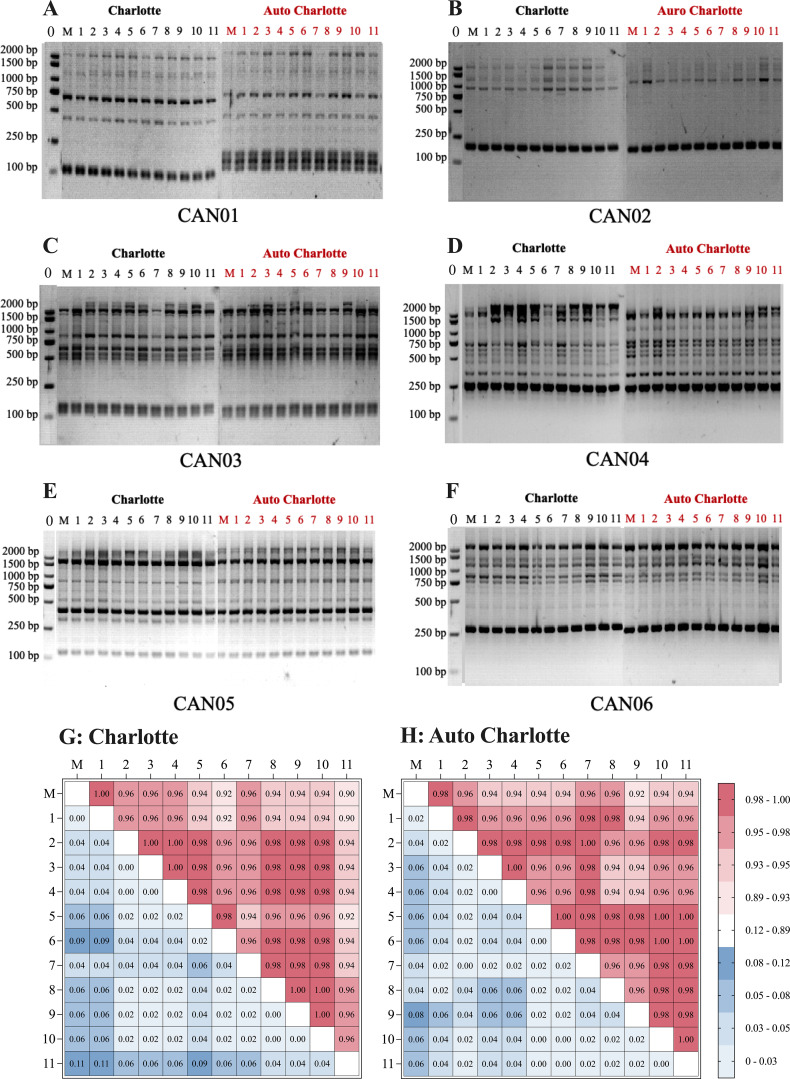
Genetic similarity analysis of cannabis plantlets from different batches based on ISSR markers. **(A–F)** Electrophoretic profiles amplified by six ISSR primers. Lane 0: DL2000 DNA Marker (Vazyme Biotech Co., Ltd., Nanjing, China); Lane M: mother plant; Lane 1-11: plantlets from different harvest batches. **(G, H)** Matrices of Nei’s genetic identity (red) and genetic distance (blue) for ‘Charlotte’ and ‘Auto Charlotte’, respectively.

Six ISSR primers with stable amplification and clear banding patterns were selected for analysis. Using these primers, 49 and 52 distinct bands were amplified in ‘Charlotte’ and ‘Auto Charlotte’, respectively (**Figures 5A–F**). Based on the binary presence/absence matrix (**Supplementary Figure 5**), Nei’s genetic identity and genetic distance were calculated among all samples (**Figures 5G, H**). For both cultivars, Nei’s genetic identity between plantlets from all batches and their corresponding mother plants exceeded 0.90, with genetic distances were generally below 0.10. These results indicate a high level of genetic fidelity between the plantlets of all batches and their mother plants.

### 3.3 Simulating the effect of mother plant culture cycle on annual production

**Figure 6:**
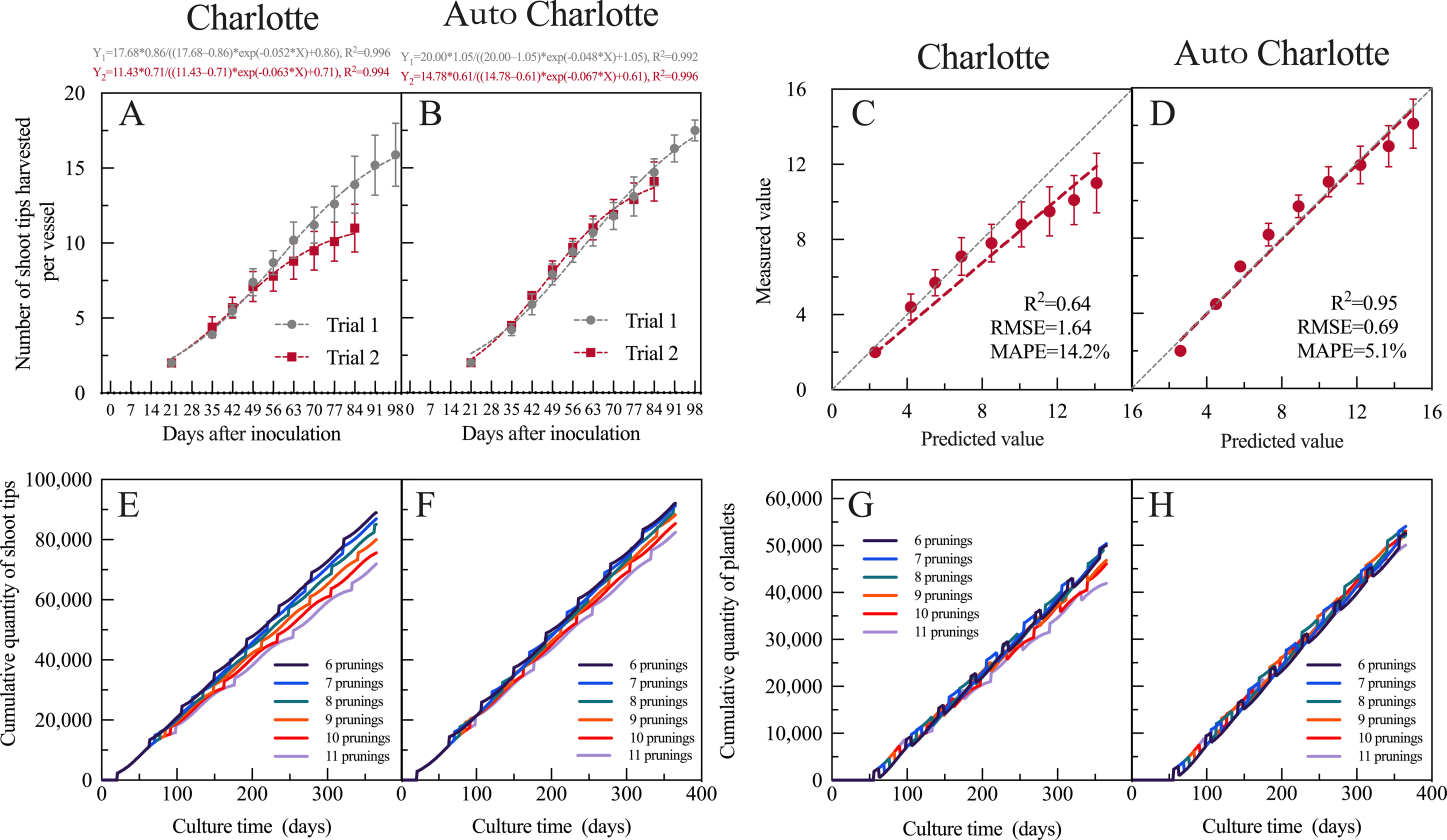
Effects of mother plant culture cycle on annual propagation production. **(A, B)** Cumulative shoot tip yield and logistic model fitting for ‘Charlotte’ and ‘Auto Charlotte’ at an inoculation density of two mother plants per vessel. **(C, D)** Model validation results, including the coefficient of determination (R^2^), root mean square error (RMSE), and mean absolute percentage error (MAPE). The predicted values were calculated from the fitted model equation based on the first trial data, while the measured values were obtained from the second trial. **(E, G)** Annual cumulative yield of shoot tips and plantlets for ‘Charlotte’ under different culture cycles were obtained via model simulation; **(F, H)** the corresponding results for ‘Auto Charlotte’. Numbers 6, 7, 8, 9, 10 and 11 in the legend indicate the number of mother plant harvesting batches within a single culture cycle.

Cumulative shoot tips production in both trails was well described by the logistic growth model, with *R^2^* exceeding 0.99 for both cultivars (**Figures 6A, B**). However, in the second trial, both the number of harvested per vessel and the harvest batches were lower than the first trial. Specifically, ‘Charlotte’ and ‘Auto Charlotte’ produced 11.0 and 14.1 shoot tips per vessel over nine batches, respectively, compared with 15.9 and 17.5 shoot tips over 11 batches in the first trial. This reduction was likely attributed to variations in harvest intensity and liquid medium supplementation in the second trial, which negatively affected mother plant vigor and regenerative capacity. Model validation, performed by comparing values predicted using data from Trial 1 against measured data from Trial 2, revealed differential prediction accuracy between cultivars. For ‘Charlotte’, prediction accuracy was moderate (*R^2^* = 0.64, RMSE = 1.64, MAPE = 14.2%), whereas for ‘Auto Charlotte’ the model exhibited high accuracy (*R^2^* = 0.95, RMSE = 0.69, MAPE = 5.1%). These findings indicated that the model performed better for ‘Auto Charlotte’, while noticeable deviations were observed in the prediction for ‘Charlotte’ after the 5th batch.

A scaled-up production simulation was conducted to evaluate annual propagation capacity under different mother plant culture cycles (**Figures 6E–H**). A 98-day culture cycle (11 batches), which produced the lowest annual shoot tip yields. The values were 71, 993 and 82, 497 shoot tips m^−2^ for ‘Charlotte’ and ‘Auto Charlotte’, with corresponding commercial plantlet production of 41, 965 and 50, 069 plants m^−2^. In comparison, a shorter 70-day cycle (7 batches) greatly improved propagation efficiency. Annual shoot tip yield per unit area increased to 86, 932 for ‘Charlotte’ and 91, 394 for ‘Auto Charlotte’, representing increases of 20.8% and 10.8%, respectively. Importantly, annual plantlet production reached peak values of 50, 417 and 54, 034 plants m^−2^, corresponding to increases of 20.1% and 7.9%. Further shortening the culture cycle to 63 days (6 batches) slightly increased shoot tip harvest number but reduced annual plantlet yield. This was because more plantlets were needed for mother plant renewal, leaving fewer available for commercial production.

## 4 Discussion

### 4.1 PAM technology enhances propagation efficiency of medicinal cannabis

This study demonstrated that PAM technology significantly enhances the propagation efficiency of repeated shoot tip harvest for medicinal cannabis. Mother plants achieved 11 harvest batches within a 98-day cycle with propagation coefficients of 12.0 for ‘Charlotte’ and 11.3 for ‘Auto Charlotte’, which are far more efficient than those reported for conventional micropropagation systems (Mestinšek-Mubi et al., 2020; Ioannidis et al., 2020). PAM optimizes the *in vitro* environment by improving light utilization, air exchange and CO_2_ supply to boost plantlet physiological activity and growth rate (Kozai and Kubota, 2001; Liang et al., 2026). This is different from conventional micropropagation systems relying on exogenous sucrose, as plantlets in these systems often suffer from low CO_2_ levels, high humidity and poor ventilation. These conditions suppress photosynthesis, reduce propagation efficiency and prolong culture cycles (Murphy and Adelberg, 2021; Shi et al., 2024). By contrast, PAM provides growth conditions that more closely resemble the ex-vitro environment, enabling sustained vegetative growth vigor and prolonged shoot tip production. For instance, Kodym and Leeb (2019) reported continuous shoot tip harvesting for up to six months under PAM while maintaining stable shoot tip quality. Similarly, McKay et al. (2025b) demonstrated that PAM technology employing porous support materials supplemented with liquid medium enabled repeated shoot tip harvesting over 16 weeks, substantially improving the propagation coefficient.

In addition, PAM technology also eliminates the 1–2 weeks acclimatization period typically required in conventional micropropagation (Kozai, 2010), and enables reductions in production costs and labor (Zarei et al., 2021). In this study, plantlets cultured for 35 days via PAM were directly transplanted and exhibited rapid adaptation and vigorous growth (**Figure 4**). This observation aligns with previous reports showing that rooting and acclimatization can occur simultaneously under PAM conditions (Kodym and Leeb, 2019), driven by the enhanced photosynthetic capacity and improved stomatal function of plantlets under photoautotrophic conditions (Xiao et al., 2011). Collectively, these results support that PAM not only substantially improves propagation efficiency of the repeated harvest protocol, but also enables direct transplanting without acclimatization, thereby highlighting its great potential for large-scale and industrialized propagation of medicinal cannabis.

### 4.2 PAM ensures consistency in growth traits and high genetic similarity of cannabis plantlets

Plantlets produced via repeated shoot tip harvesting under PAM conditions exhibited high phenotypic uniformity (**Figure 4**) and strong genetic fidelity to their respective mother plants (**Figures 5G, H**). The PAM environment, characterized by relatively high light intensity, appropriate CO_2_ concentration, and moderate ventilation, substantially reduces physiological disorders commonly observed in conventional micropropagation, such as hyperhydricity, leaf malformation, and uneven shoot elongation (Kozai and Kubota, 2001; Xiao et al., 2011). Moreover, PAM substantially reduces or eliminates the need of plant growth regulators (PGRs), thereby minimizing the risk of somaclonal variation (Kodym and Leeb, 2019; McKay et al., 2025b). In this study, only 0.5 mg L^−1^ of IBA was applied during the initial rooting stage of mother plants, and no PGRs were used during subsequent culture. This low PGRs protocol promoted root development while mitigating hormone-induced genetic variation.

In our study, we similarly found high phenotypical consistency among plantlets from different batches produced via PAM technology. Although explant size decreased during continuous harvest, as evidenced by reduced stem diameter (**Figure 3E**) and biomass (**Supplementary Figure 4**) in batches 3 to 11 (with the first batch plantlets relatively larger due to strong apical dominance, which is consistent with McKay et al. (2025b), key growth indicators showed no significant declines (**Figure 3**). Growth performance assessments after transplanting further supported this conclusion. For ‘Charlotte’, plantlets from batch 1 were originally derived from apical shoots with typical apical dominance, yet plantlets from batches 5 and 11 achieved similar growth status and synchronized morphology following an additional week of culture. For ‘Auto Charlotte’, no significant differences were observed among batches after three weeks of growth. Overall, these results confirmed high growth consistency and stability across all harvest batches. These findings align with Kurtz et al. (2022), who reported that plantlets propagated via repeated shoot tip harvest exhibited plant architecture, inflorescence biomass, and cannabinoid profiles comparable to those of plants propagated through cuttings after an appropriate extension of growth time.

At the genetic level, although previous studies have reported that prolonged *in vitro* culture may induce epigenetic alterations in cannabis, such as decreased DNA methylation levels (Kastelec et al., 2025), the low environmental stress and minimal PGR use in PAM reduced the risk of such variation (Dreger et al., 2025; Kodym and Leeb, 2019). In this study, ISSR molecular marker analysis revealed extremely low genetic polymorphism and highly consistent banding patterns between mother plants and plantlets from different batches (**Figure 5**; **Supplementary Figure 5**), confirming the effectiveness of PAM in maintaining high genetic similarity. This genetic fidelity is essential for standardized, large-scale production of medicinal cannabis plantlets with consistent chemical profiles.

### 4.3 Optimization of production cycle to maximize annual cannabis plantlet propagation yield

Numerous studies have supported that tissue culture technology combined with appropriate propagation protocols can substantially increase cannabis propagation efficiency compared with cutting propagation (Wongchaya et al., 2025; Lubell-Brand et al., 2021). For instance, Lubell-Brand et al. (2021) demonstrated that 2, 000 shoot tips could be harvested per m^2^ of culture area over a 10-week culture cycle, with the propagation yield reaching nine times that of cutting propagation. In this study, the MP2 treatment achieved the highest shoot tip yield per vessel (**Figures 2C, D**). Over a 98-days culture cycle, the shoot tip yields of ‘Charlotte’ and ‘Auto Charlotte’ reached 2833 and 3115 per m² of culture area, respectively, with the propagation rate per unit time and area also being nine times that of cutting propagation (Lubell-Brand et al., 2021). Further comparison revealed that our propagation protocol shared a similar protocol with that reported by McKay et al. (2025a). They achieved five batches of shoot tip harvests within 15 weeks, and observed gradual reductions in shoot tip length and dry weight over successive batches. This decline is presumably attributed to the relatively high mother plant inoculation density, which promotes excessive branching, induces mutual shading and insufficient light capture, and consequently suppresses shoot tip growth over successive batches. Similarly, in this study, an excessively high mother plant inoculation density (MP3) reduced growth space and survival rate (**Supplementary Figures 2**, **3**), failing to enhance the shoot tips yield per unit area. As confirmed by **Supplementary Table 2**, inoculation density significantly affected mother plant survival rate in the later culture period. Thus, optimizing inoculation density of mother plants is important for improving shoot tip yield per unit area.

Given the high propagation coefficient and genetic similarity, optimizing the culture cycle of mother plants is particularly critical for maximizing annual shoot tip yield (Liang et al., 2024). In our study, we applied cultivar-specific logistic growth model to predict the annual yields under different mother plant culture cycles. Results showed that the annual shoot tip yield of both cultivars under the 70-day culture cycle were significantly higher than those under the 98-day cycle (**Figures 6G, H**). Therefore, it is recommended a 70-day (seven batches) mother plant culture cycle to maximize the annual propagation yields of cannabis plantlets.

Notably, the PAM system exhibits high environmental sensitivity, which necessitates stringent precision in operational management. This was evidenced in the second trial, where the number of shoot tip harvest batches for both cultivars decreased to nine, accompanied by a reduced shoot tip yield in ‘Charlotte’ (**Figures 6A, B**). This discrepancy may be attributed to two factors: (1) reduced vegetative growth vigor following intensive harvesting in the early stage, and (2) inappropriate liquid medium supplementation volume after each harvest. Excessive supplementation may lead to waterlogging and root hypoxia, whereas insufficient supplementation may impose drought stress. In addition, suboptimal mineral nutrition and ion ratios in the liquid medium may also impair plant growth stability and propagation efficiency (Akin et al., 2020; Kovalchuk et al., 2018). Consequently, in practical production, synergistic optimization of harvest intensity, liquid medium supplementation and mineral nutrient composition is essential to maintain long-term mother plant vigor and ensure stable, high-quality shoot tip production. This study establishes an efficient propagation system producing medicinal cannabis plantlets with high genetic similarity and fine quality, and provides a reference for standardized *in vitro* propagation of other medicinal and economic plant species.

## 5 Conclusion

This study established an efficient PAM-based propagation protocol for medicinal cannabis, enabling continuous and stable production of all-female plantlets with high genetic similarity. Mother plants achieved 11 harvest batches within a 98-day cycle. Regenerated plantlets of both cultivars exhibited consistent morphological and physiological traits, with rooting rates exceeding 70%. ISSR marker analysis confirmed high genetic fidelity, with Nei’s genetic identity coefficients above 0.90 between plantlets and their respective mother plants. Notably, these plantlets could be directly transplanted without acclimatization. The cultivar-specific logistic growth model indicated that a 70-day culture cycle for mother plants maximized propagation yield. Future optimization of key factors such as liquid medium supplementation and pruning strategies is expected to further improve the propagation efficiency and plantlet quality.

## Data Availability

The original contributions presented in the study are included in the article/**Supplementary Material**. Further inquiries can be directed to the corresponding author.

## Supplementary material

Supplementary Table 1 ISSR primers for genetic similarity analysis.

Supplementary Table 2 ANOVA results for effects of inoculation density and cultivar on plant performance over time in the PAM system.

Supplementary Figure 1 Spectral distribution of white LED lamps.

Supplementary Figure 2 Growth status of mother plants throughout the culture cycle.

Supplementary Figure 3 Changes in the survival rate of mother plants under different inoculation densities.

Supplementary Figure 4 Biomass of plantlets from different batches.

Supplementary Figure 5 The 0/1 matrix diagrams of amplification products from mother plants and plantlet samples using different primers.
