# Supplementary material for: Efficient propagation protocol and genetic similarity assessment of medicinal cannabis based on photoautotrophic micropropagation
Source: Front Plant Sci. 2026 Jun 10;17:1846572. doi: 10.3389/fpls.2026.1846572 (PMC13290919; doi:10.3389/fpls.2026.1846572)
Supplement: Supplementary Table 1 — ISSR primers for genetic similarity analysis. [file DataSheet1.docx]

Supplementary Material

# Efficient propagation protocol and genetic similarity assessment of medicinal cannabis based on photoautotrophic micropropagation

Juwen Liang^1,3†^, Xiuye Wei^2†^, Jun Liu^1,3^, Qing Zhou^1,3^ and Dongxian He^1,3,4^*

**Supplementary Table 1. ISSR primers for genetic similarity analysis.**

| Primer | Repeat | Forward primer sequence (5'-3') | Reverse primer sequence (5'-3') | References |
| --- | --- | --- | --- | --- |
| CAN01 | (AAG)7 | AGGAACACTTTGAAAGCGAG | CGGTCATCTACCTTGAGCTT | Xin, 2015 |
| CAN02 | (AG)9 | GGACAACACAGAAATGAGCA | CACTTTGCAGCCTATTTCCT |  |
| CAN03 | (CTA)9 | CAAATGCCACACCACCTTC | GTAGGTAGCCAGGTATAGGTAG | Adamek et al., 2023 |
| CAN04 | (TTG)9 | TTGATGGTGGTGAAACGGC | CCCCAATCTCAATCTCAACCC |  |
| CAN05 | (GTT)7 | GGTTGGGATGTTGTTGTTGTG | AGAAATCCAAGGTCCTGATGG |  |
| CAN06 | (ACG)7 | TGGTTTCAGTGGTCCTCTC | ACGTGAGTGATGACACGAG |  |


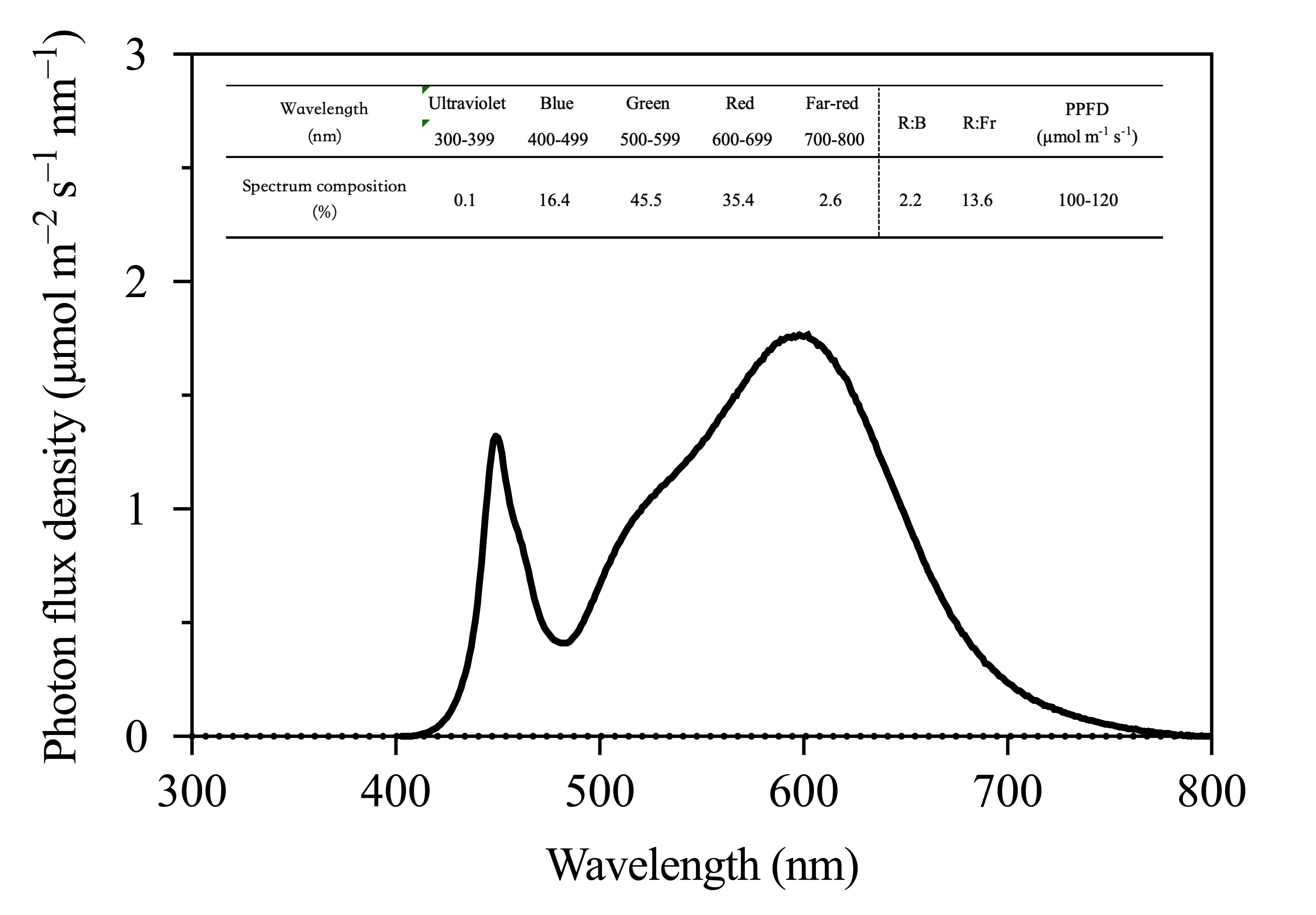


**Supplementary Figure 1. Spectral distribution of white LED lamps.** The spectral distribution across the 300~800 nm wavelength range was quantified using a fiber optic spectrometer (AvaSpec-ULS2048, Avantes Inc., the Netherlands). The spectral composition was as follows: 0.1% ultraviolet light (300~399 nm), 16.4% blue light (400~499 nm), 45.5% green light (500~599 nm), 35.4% red light (600~699 nm), and 2.6% far-red light (700~800 nm).

**Supplementary Table 2. ANOVA results for effects of inoculation density and cultivar on plant performance over time in the PAM system.**

| Measurements |  | Days after inoculation | | | | | | | | | | |
| --- | --- | --- | --- | --- | --- | --- | --- | --- | --- | --- | --- | --- |
|  |  | 21 | 35 | 42 | 49 | 56 | 63 | 70 | 77 | 84 | 91 | 98 |
| Mother plant survival rate | I | NS | * | * | ** | NS | NS | NS | NS | ** | ** | ** |
|  | C | NS | * | * | ** | * | * | * | * | NS | NS | NS |
|  | I × C | NS | * | * | ** | NS | * | * | * | NS | NS | ** |
| Shoot tip yield per vessel per harvest | I | ** | * | ** | * | NS | NS | NS | NS | NS | NS | NS |
|  | C | NS | * | * | NS | NS | NS | NS | NS | NS | NS | NS |
|  | I × C | NS | NS | * | NS | NS | NS | NS | NS | NS | NS | NS |
| Cumulative yield per vessel | I | ** | ** | ** | ** | ** | ** | ** | ** | ** | ** | ** |
|  | C | NS | NS | * | NS | NS | NS | NS | NS | NS | NS | NS |
|  | I × C | NS | NS | NS | NS | NS | NS | NS | NS | NS | NS | NS |
| Propagation coefficient | I | * | ** | ** | ** | ** | ** | ** | ** | ** | ** | ** |
|  | C | NS | NS | * | NS | NS | NS | NS | NS | NS | NS | NS |
|  | I × C | NS | NS | NS | NS | * | NS | NS | NS | NS | NS | NS |

Abbreviations are defined as follows: I denotes mother plant inoculation density; C denotes cultivar; I×C represents their interaction effect. Statistical significance levels are marked with * for p < 0.05 and ** for p < 0.01 (n = 12); NS indicates no significant difference.


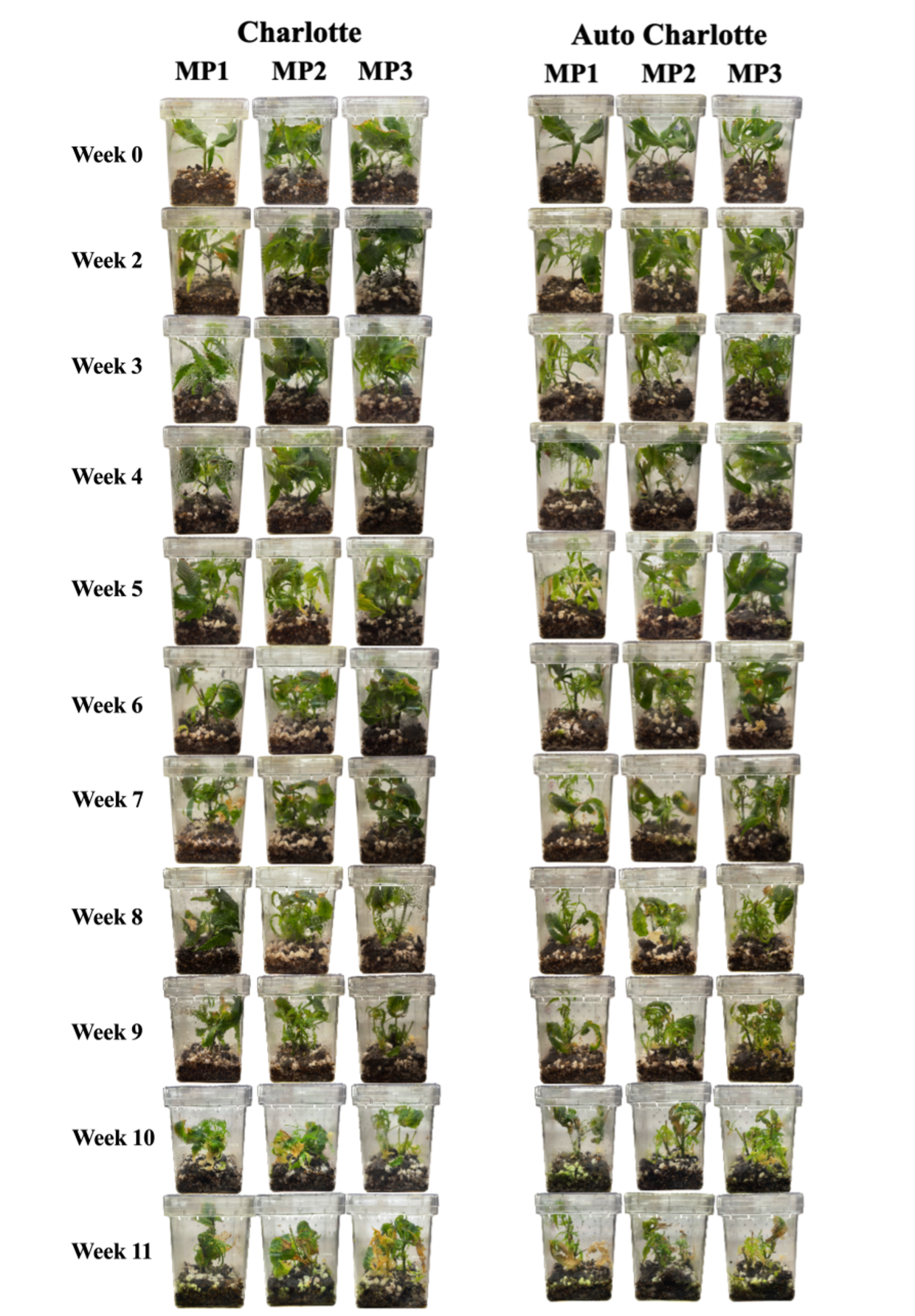


**Supplementary Figure 2. Growth status of mother plants throughout the culture cycle.** Among them, MP1, MP2, and MP3 represent the number of mother plants inoculated per vessel, which are 1, 2, and 3 plants, respectively. Week 0, Week 2, ..., Week 11 indicate the number of weeks after the first harvest.


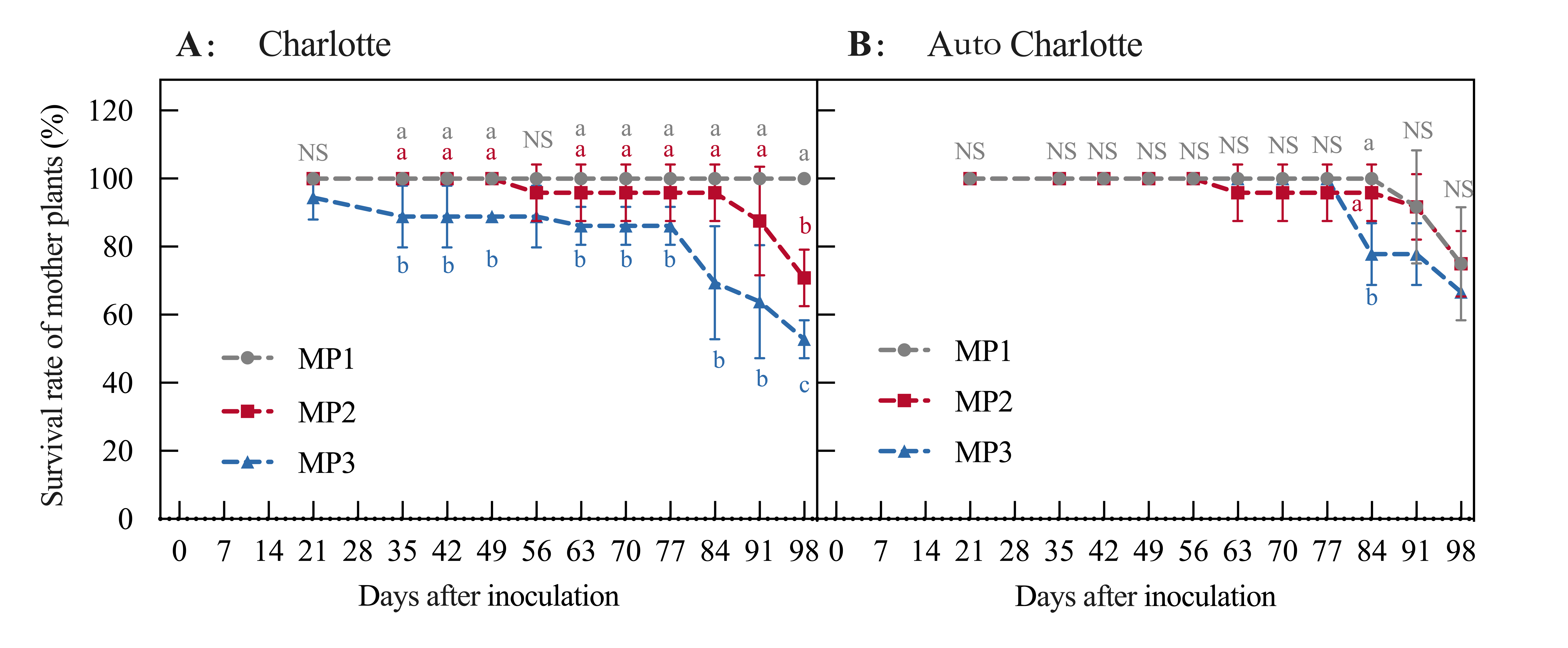


**Supplementary Figure 3. Changes in the survival rate of mother plants under different inoculation densities.** (A, B) Results for the ‘Charlotte’ and ‘Auto Charlotte’ cultivars, respectively. Among them, MP1, MP2, and MP3 represent the number of mother plants inoculated per container, which are 1, 2, and 3 plants, respectively. Different letters indicate significant differences (*P* < 0.05, n = 12). NS denotes no significant difference.


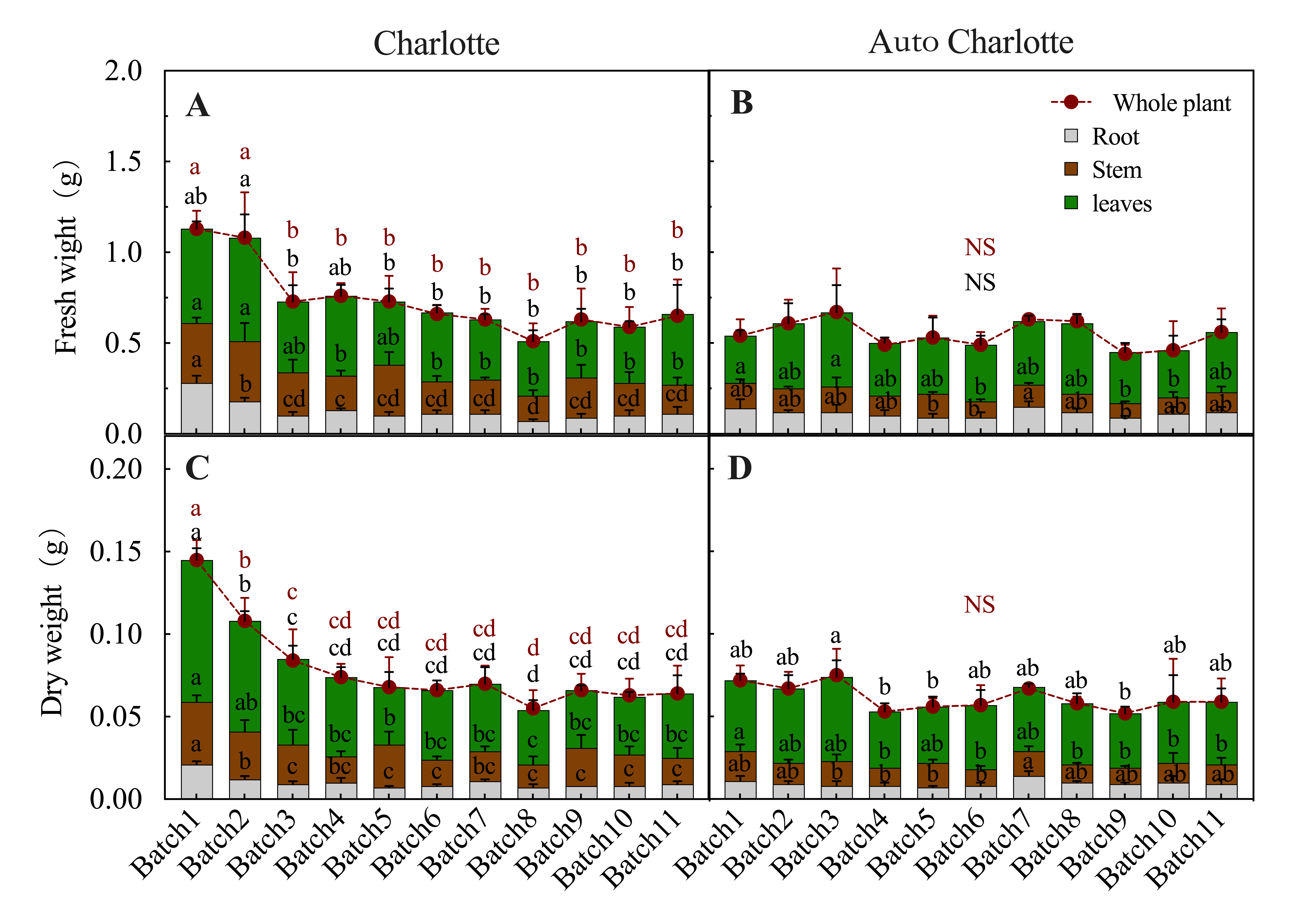


**Supplementary Figure 4. Biomass of plantlets from different batches.** (A, C) The fresh weight and dry weight of roots, stems, leaves, and whole plants of ‘Charlotte’ plantlets across batches; (B, D) The corresponding indicators of ‘Auto Charlotte’ plantlets. Different letters indicate significant differences (*P* < 0.05, n = 4). NS denotes no significant difference.


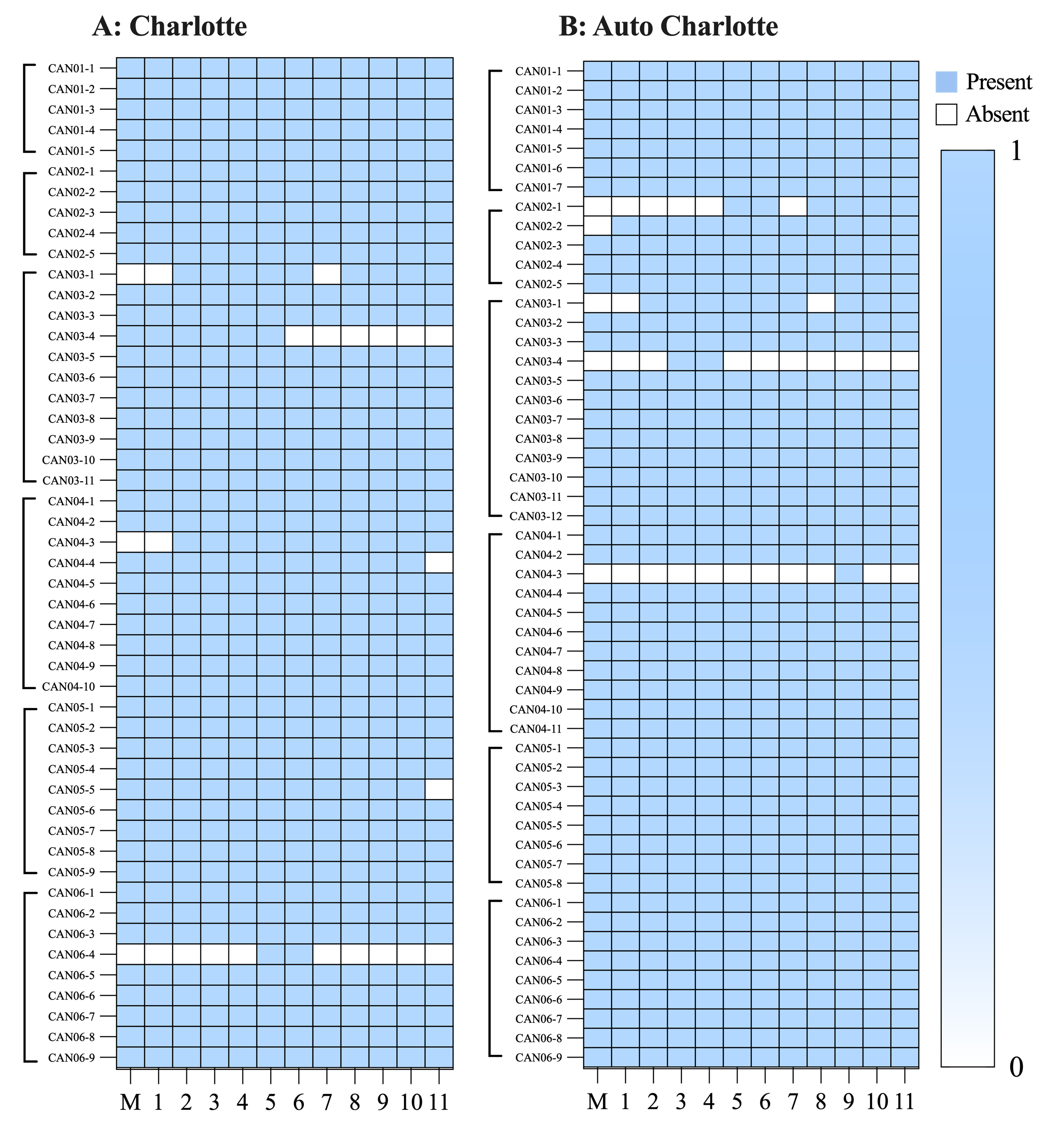


**Supplementary Figure 5. The 0/1 matrix diagrams of amplification products from mother plants and plantlet samples using different primers.** (A, B) The results for ‘Charlotte’ and ‘Auto Charlotte’ cultivars, respectively. Among them, CAN01, CAN02, CAN03, CAN04, CAN05, and CAN06 represent the primer names.
